# Preventable Burns from Domestic Tap Water

**DOI:** 10.3390/ebj3020031

**Published:** 2022-05-09

**Authors:** Max Prokopenko, Alistair J. M. Reed, Maria Chicco, Fadi Issa

**Affiliations:** 1 Department of Plastic and Reconstructive Surgery, Stoke Mandeville Hospital, Buckinghamshire Healthcare NHS Trust, Aylesbury HP21 8AL, UK; alistair.reed@nhs.net (A.J.M.R.); maria.chicco2@nhs.net (M.C.); 2 Nuffield Department of Surgical Sciences, University of Oxford, John Radcliffe Hospital, Oxford OX3 9DU, UK

**Keywords:** burns, scalds, thermostatic mixing valves

## Abstract

Tap water scalds from domestic outlets can afflict large body surface areas. Such injuries are preventable and carry significant associated morbidity, mortality, and economic burden. Previously identified risk factors include age (<5 or >65 years old) and the presence of physical or mental disabilities. Education campaigns and advances in legislation mandating the restriction of tap water temperature at user outlets have been employed in an attempt to prevent such injuries. Nonetheless, the incidence of these injuries persists, and further mitigating measures must be implemented to minimize their occurrence. The purpose of this study was to determine the groups at risk for such injuries and whether this has recently changed. A retrospective observational study was carried out to include patients admitted with tap water scalds to a regional burn’s unit from October 2016 to September 2020. Twenty-three patients were included, and their incidence was 5.75 cases per year, equating to 5.1% of all scalds requiring inpatient treatment. The very young (<5 years old) and elderly (>65 years old) accounted for the majority of admissions (65.2%), 26.1% had a mental disability, and 30.4% had a physical disability. Tap water scalds continue to cause preventable injuries affecting all ages, and in particular, the elderly and patients with pre-existing disabilities.

## 1. Introduction

Domestic tap water scalds have been a recognised public health hazard since the 1970s [1] and occur as a consequence of splashes, spills, or immersions. Larger burns are associated with significant morbidity and mortality and carry a high socioeconomic burden. Previous studies based in the United States have estimated the cost at USD 113,000 for inpatient hospital treatment in those over the age of 60 [2]. This figure excludes the additional cost of outpatient appointments and longer-term treatment and rehabilitation. The incidence of domestic hot tap scalds is higher amongst the most vulnerable in society, including the very young, elderly, those with psychomotor disabilities, and those belonging to lower socioeconomic backgrounds [1,3,4,5]. This is further supported by a study previously carried out in our own unit, demonstrating that the very young and adults with pre-existing psychomotor disabilities are most commonly affected [6]. Such injuries are potentially preventable, and further measures should be introduced to reduce the risk of sustaining tap water scalds in the domestic setting, especially for those at greatest risk.

Historically, hot tap water has been maintained at ≥60 °C to prevent the growth of Legionella pneumophila [7]. However, at this temperature, water can cause deep-dermal burns after three seconds and full-thickness burns after five seconds in adults, with even shorter exposures or lower temperatures producing similar burns in children [8]. Vulnerable individuals with longer reaction times are therefore at higher risk of domestic tap water and immersion burns. The elderly, in particular, are significantly affected by tap water scalds. Poor sensorimotor perception, slower reaction times, poorer mobility, frailty, and cognitive decline predispose this population to injury [3,9,10,11].

Immersion burns may be entirely preventable. Legislation to reduce household water outlet temperature has demonstrated a positive impact in previous American and Australian studies [12,13] These population-based studies indicate a decline in the rate of hospitalisations per year following scald injury after the changes in legislation. One method of restricting water temperature at household outlets is to use thermostatic mixing valves. These enable stored water to be maintained at higher temperatures whilst limiting the temperature of water at the outlets. In the United Kingdom (UK), building regulations updated in 2010 necessitated a hot water temperature limit of 48 degrees Celsius at outlets in order to prevent scalds by the use of appropriate temperature control devices, including thermostatic mixing valves (TMVs) [14]. However, this is limited to new homes only and does not apply to homes built prior to the implementation of the legislation [15].

The purpose of this study was to review the incidence and aetiology of patients admitted with domestic tap water scalds. Despite changes in legislation, and the introduction of new rules to limit domestic water temperature at outlets, we hypothesise domestic tap water scald injuries will continue to be a prevailing issue with the greatest impact on the most vulnerable patients. 

## 2. Materials and Methods

This is a retrospective observational study from a district general hospital (DGH) located in the United Kingdom, which has an on-site specialist burns service and burns unit. This study included all patients admitted at any age with domestic tap water scalds from October 2016 to September 2020. 

Domestic hot tap water burns were defined as those occurring in a domestic setting caused by water directly from a hot tap or shower (as opposed to water heated by an appliance, such as a kettle). Burn depth was defined as superficial partial-thickness (SPT), mid-dermal (MD), deep dermal (DD), or full-thickness (FT). Burns containing both superficial and deeper components were categorised as mixed depth.

Data for the patients admitted to our unit were collected from a prospectively maintained local database. The database was screened for all inpatient scald burns treated in our unit in the defined time period using codes: Code 04—scald; NO6—tap splash/spill; Q01—bathing immersion; Q02—sink immersion; and Q07—shower. Data included patient demographics, the nature and cause of injury, place of injury and whether it was witnessed, co-morbidities and disabilities, burn site, depth, and the calculated total body surface area (TBSA) % affected. Furthermore, we collected data on the management of each case, including whether patients underwent resuscitation on admission, if the injury was managed non-operatively or operatively, and whether the burns required skin grafting. We report on patient outcomes, including the time taken for burns to heal, the length of stay, mortality, complications, and the requirement for further surgical procedures. We performed further subgroup analysis for patient age and burn size.

We defined the vulnerable age groups as <5 years and >65 years, and psychomotor impairment was defined as the presence of a disorder of the mind or cognitive function which could lead to impaired perception of hazards, and or delayed or no reaction in response to the stimulus sustained from the injury. Mobility impairment was defined as any degree of physical disability of the upper or lower limbs resulting in diminished dexterity, coordination, or power and included both congenital and acquired conditions.

## 3. Results

### 3.1. Demographics

During the 4-year period from October 2016 to September 2020, our unit admitted a total of 23 patients with domestic tap water scald injuries, of which 11 were male and 12 were females, equating to 5.75 admissions per year. This accounted for 5.1% (23/454) of all the scald injuries requiring inpatient treatment. The very young (<5 years) and elderly (>65 years) age categories accounted for the majority of admissions, representing 4 (17.4%) and 11 (47.8%) cases, respectively. The mean age was 48.8 ± 31.6 (range 1–90). Baseline demographics are given in Table 1.

A total of 16 out of 23 cases had underlying co-morbidities. Six cases (26.1%) had some form of psychomotor impairment. These included patients with autism, cerebral palsy, two cases with dementia, poorly controlled epilepsy, and cerebral metastatic disease. Furthermore, of the 23 patients, 7 (30.4%) had documented impaired mobility due to spinal cord injury, cerebral palsy, joint arthroplasty with foot drop, and autoimmune arthropathies. Finally, a single patient had formal diagnoses of peripheral neuropathy and partial sightedness, conditions causing sensory deficits. Other co-morbidities recorded included alcohol excess, diabetes mellitus, glaucoma, gout, hypertension, hypothyroidism, psoriatic arthritis, and ulcerative colitis. Four patients had either formal carers or the next of kin was the main carer. One patient lived in supported accommodation. None of the patients were nursing home residents.

### 3.2. Burn Site

Most burn injuries involved more than one anatomical region (69.6%). 18 (78.3%) cases had burns involving the lower half of the body (Table 2). The buttocks were the single most frequently involved area, with eight cases (34.8%) presenting with a burn in this area, followed by the foot and ankle with six cases (26.1%). The head, back, chest, and perineum were the least frequently involved and were found to be involved only once each (4.3%). Injury characteristics are given in Table 2.

### 3.3. Burn Size

The range of burn size was from 0.4–43% of the total body surface area (TBSA). The mean size of burns was 9.4% TSBA (±10.2). Mean %TBSA across the four cases who were <5-years old was 8.75%, with one of these cases sustaining 25% TBSA burns. In the >65-year-old age bracket, the mean burn size was 6.7% TBSA, with the single largest being 19.5%. There was a single mortality in our cohort, with a 43% TBSA burn.

### 3.4. Burn Depth

Approximately half of the cohort (52.2%) sustained superficial burns only. A further four cases had deep-dermal burns (17.4%). Two cases sustained a combination of deep dermal to full-thickness burns. Mixed depth burns (superficial and deep) were reported in two cases, and the burn depth was unrecorded in three of the patients. Of the four very young (<5 years old) cases in our cohort, three sustained only superficial burns, with the fourth case having the burn depth unrecorded. In patients over 65-years old, a single case sustained deep-dermal to full-thickness burns, three cases of deep-dermal burns and a single case of mixed-depth burns. The single mortality in this cohort suffered from burns assessed as deep-dermal to full-thickness in depth.

### 3.5. Cause of Injury

Bathing immersion was the most common cause of injury, resulting in 18 (78.3%) cases over the four-year period. Three (13.0%) cases were secondary to scalds directly from the hot tap, and two (8.7%) were due to shower scalds. Most injuries (73.9%) took place at home; however, six cases occurred at a relative’s home (13.0%), or at a hotel or hostel (13.0%).

Overall, most burns in this cohort were unwitnessed injuries (82.6%). Of the four children in the <5-year-old group, three were unwitnessed. Two cases sustained a burn after turning on a hot water tap, and two cases sustained burns from immersion in hot bath water by climbing into the bath. In the other age subgroups, a further total of 16 (69.6%) cases were unwitnessed. There were two shower scalds, one of which was due to the partner showering the patient, and the other was secondary to a loss of consciousness and collapse. The other 16 (69.6%) adult cases were burns due to bathing immersion. Six cases were scalded when adding water from the hot tap whilst bathing.

In four cases, the patients themselves or their carers were unaware of the water temperature in the bath. Two of these were unable to recognise the temperature of the water due to underlying co-morbidities: one was paraplegic, and the other was insensate with peripheral neuropathy. One patient was hoisted into a hot bath by carers. Five patients fell into a bath. One was due to a seizure on a background of poorly controlled epilepsy. Another was secondary to intoxication with alcohol. The other three cases slipped and fell into the hot bath water and were unable to get out. One patient with documented cerebral metastases and vascular dementia was found in a hot bath by their partner.

### 3.6. Burn Injury Management

**Figure 1:**
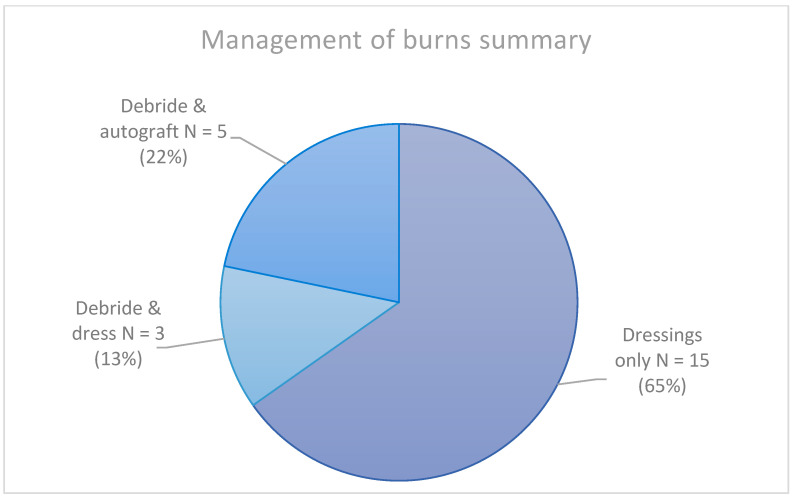
Summary of management of tap water scalds admitted to our unit.

Of the 23 patients admitted to our unit, 15 (65.2%) were managed non-operatively with dressings only. The other eight (34.8%) patients were managed operatively. Of these, three patients requiring debridement and dressing (13.0%), one case which was a simple dressing, and the other two biological dressings (Biobrane™). The remaining five underwent debridement and resurfacing with auto-graft. Seven (30.4%) of the admitted patients required intravenous fluid resuscitation on admission. See Figure 1.

### 3.7. Patient Outcomes

The mean length of stay was 13.8 ± 17.6 days, ranging from 1 to 66 (Table 3). Of the 23 patients in this cohort, the time taken for burns to fully heal was documented in 13 cases (56.5%), and calculated to be 32 ± 39.1 days, ranging from 5–129 days. One patient with 43% body surface area deep-dermal to full-thickness burns died one day after admission following a cardiac arrest. The modified Baux score for this patient was 71. Two patients required surgical re-intervention at a later stage. One of these patients with mixed depth 10% TBSA burns was managed initially with dressings alone but required re-admission with infected burns three days post-discharge and underwent debridement and auto-graft. Another case in the <5-years old age group with 25% body surface area burns was managed with debridement and auto-graft and required further debridement and auto-graft a month later for unhealed burns.

## 4. Discussion

This study confirms domestic tap water scalds continue to cause significant morbidity and mortality despite changes in legislation to limit the water temperature at domestic outlets. Furthermore, vulnerable individuals, such as those with disabilities, co-morbidities, or psychomotor impairment, appear to be at a higher risk of injury. A previous study undertaken in our unit investigated hot bath water scalds between 1990 and 1997 [6], demonstrating tap water scalds to be life-threatening injuries that predominantly affect inadequately supervised children and adults with pre-existing psychomotor disabilities in the domestic environment. In our study, eighteen patients sustained immersion burns, accounting for 0.5% of all burns seen by our unit over the time period investigated, compared with a rate of 3.8% seen previously in the 1990s.

Between the two studies from our unit, there appears to be a shift in the age demographic affected from the very young to a more elderly cohort. Our data demonstrate a total of five children (21.7%) with such injuries, representing a minority of the cohort compared with the elderly, who made up almost half of all patients requiring admission. In contrast to the data presented by Cerovac et al., children under 4 years old represented the single largest group, with approximately two-thirds of all patients being children. Comparing our admission data with that presented by Cerovac et al., the number of adult admissions to our unit per annum rose from 2.25 to 4.75, whilst the number of paediatric cases fell from 4.88 to 1. Future policies and measures intending to mitigate domestic tap water scalds should be particularly considerate of the more elderly population group, who are particularly at risk of such injury.

A direct comparison with the data presented by Cerovac et al. is limited due to the subsequent development of the UK National Network for Burns Care. The guidelines produced resulted in the triage of burns injuries to burns facilities, units, and centres according to the size, type, depth, and severity of the injury. As a result, the cohort of patients presenting to our unit may not be due to true changes in population incidence. Furthermore, the UK population size and age structure have changed between the time periods of when the studies were carried out. The estimated population has increased by approximately 10 million and the number of those aged 65 years and over is growing faster than those under 65 years, indicating an ageing population [16].

The mean burn size and affected anatomical site found in our study are comparable to that described in the literature, with the most frequently burnt areas being the lower limbs and buttocks [1,2,17,18,19,20]. Additionally, these injuries appear to most commonly occur at home and are predominantly in the very young or elderly population groups and in those with underlying co-morbidities [2,6,18,20]. Prevention of domestic tap water scalds has previously been attempted through passive and active interventions via educational, legislative, and technological measures. Previous studies indicate that educational interventions have limited success in reducing burn injuries [21]. Passive measures, such as lowering of the domestic thermostat, or installation of thermostatic mixing valves at user outlets appear to be more effective [22]. Policy changes in New York City restricting water heater temperature to 49 degrees Celsius in dwellings constructed or renovated after 1997 demonstrated that, following policy introduction, all subsequent domestic tap water scald injuries were found in older households exempt from this new rule [20]. This is further supported by population-based studies demonstrating the effectiveness of introducing regulations to control water temperature at domestic outlets [12], with a subsequent reduction in the number of cases. The UK building regulations (updated in 2010) state that only new buildings should have temperature control devices limiting water temperature at outlets to 48 °C [14]. However, older buildings are not covered by legislation. Given the effectiveness of thermostatic mixing valves in preventing domestic tap water scalds, we propose that they should be mandatory in all dwellings, both old and new.

We recognise our study had several important limitations. Firstly, we conducted a retrospective review of cases and are limited by the small sample size and observational data. We were unable to directly compare our data with the previous study from our unit due to changes in the referral network in the UK and it is not possible to draw any conclusions on whether new building regulations played a part in reducing the incidence of domestic tap water scalds. To investigate this further, a prospective multicentre cohort study and cross-sectional study of the IBID database could provide further data on the incidence, aetiology, and treatment of domestic hot tap water burns. Additionally, further observational studies should investigate whether such injuries were sustained in households with or without water temperature control devices. Such data may shed further light on the effectiveness of legislation in preventing these injuries, although it remains clear from this study that the incidence of these injuries remains problematic.

## 5. Conclusions

Domestic tap water scalds persist as an important and preventable cause of burn injury. All age groups remain vulnerable to sustaining such injuries, however, the very young, elderly and those with underlying co-morbidities are the particular at-risk groups. Immersion burns generally tend to occur in the domestic environment and involve large areas of the body, and consequently are associated with poorer outcomes and greater morbidity and mortality. Our findings confirm that there is unacceptable stagnation in attempting to reduce the number of these preventable yet serious domestic injuries, with further work required to develop and implement preventative measures. Legislative change to enforce the installation of thermostatic mixing valves across all households nationwide represents one such strategy that we propose must be introduced to address this avoidable risk.

## Figures and Tables

**Table 1:** Patient demographics.

Variable	Frequency Total Number Patients *n* = 23	%
Age subgroups		
0–5	4	(17.4)
6–17	1	(4.3)
18–30	4	(17.4)
31–65	3	(13.0)
>65	11	(47.8)
Age (years)		
Mean (±SD)	48.8 (±31.6)	
Median	58	
Range	1–90	
Gender		
Male	11	(47.8)
Female	12	(52.2)
Co-morbidities		
Any co-morbidity	16	(69.6)
Psychomotor impairment	6	(26.1)
Mobility impairment	7	(30.4)

**Table 2:** Injury characteristics.

Variable	Frequency	%
Burn size (% TBSA)		
Mean (±SD)	9.4 (±10.2)	
Range	0.4–43	
Burn size subgroups (% TBSA)		
<1	1	
1–5	10	
6–10	5	
11–20	4	
>20%	3	
Depth of burn		
Superficial	12	(52.2)
Deep dermal	4	(17.4)
Deep dermal-full thickness	2	(8.7)
Mixed depth	2	(8.7)
Unknown	3	(13.0)
Burn injuries		
Unifocal	7	(30.4)
Multifocal	16	(69.6)
Site of burn		
Head	1	(4.3)
Back	1	(4.3)
Chest	1	(4.3)
Perineum	1	(4.3)
Buttocks	8	(34.8)
Thigh	4	(17.4)
Leg	4	(17.4)
Foot and ankle	6	(26.1)
Arm	2	(8.7)
Forearm	4	(17.4)
Hand	4	(17.4)
Cause of injury		
Scald directly from tap water	3	(13.0)
Bathing immersion burn	18	(78.3)
Shower scald	2	(8.7)
Place of injury		
Home	17	(73.9)
Relative’s home	3	(13.0)
Hotel or hostel	3	(13.0)
Witnessed/unwitnessed		
Witnessed	3	(13.0)
Unwitnessed	19	(82.6)
Unknown	1	(4.3)

**Table 3:** Patient outcomes.

Variable	Frequency	%
Time taken to heal (days)		
Mean (SD)	32 (39.1)	
Range	5–129	
Length of stay (days)		
Mean (SD)	13.8 (17.6)	
Range	1–66	
Fatality	1	(4.3)
Re-admission	1	(4.3)
Complications		
Wound infection	1	(4.3)
Unhealed burns	1	(4.3)
Further procedures		
Debridement and skin graft	2	(8.7)

## Data Availability

Data are available on request.
